# Underutilized but Sustainable: The Case for Fava Beans in the Iberian Peninsula

**DOI:** 10.3390/nu18030510

**Published:** 2026-02-02

**Authors:** Jazmín Osorio, Marta W. Vasconcelos, Elisabete Pinto

**Affiliations:** 1 Universidade Católica Portuguesa, CBQF—Centro de Biotecnologia e Química Fina—Laboratório Associado, Escola Superior de Biotecnologia, Rua Diogo Botelho 1327, 4169-005 Porto, Portugal; jperez@ucp.pt (J.O.); mvasconcelos@ucp.pt (M.W.V.); 2 EPIUnit—Instituto de Saúde Pública, Universidade do Porto, 4050-091 Porto, Portugal

**Keywords:** fava beans, legume consumption, Iberian Peninsula, plant-based proteins, consumers’ perception

## Abstract

**Background/Objectives:** Legumes, a significant source of plant-based protein, play a crucial role in diets across Portugal and Spain, contributing to both human and animal nutrition. As plant-based diets gain traction, various legumes like chickpeas, lentils, and beans have risen in popularity. However, fava beans remain underutilized compared to these varieties. This study explores stakeholder perspectives on the factors influencing the lower consumption rates of fava beans in the Iberian Peninsula, despite their nutritional and environmental benefits. **Methods:** An exploratory qualitative study was conducted using semi-structured interviews with diverse stakeholders, including nutritionists, retailers, farmers, catering professionals, and both vegetarian and non-vegetarian consumers in Portugal and Spain. **Results:** Our findings highlight a perceived lack of visibility of fava beans in supermarkets and on influential social media platforms, which often shape consumer preferences. Seasonal availability further contributes to the limited consumption, as people tend to purchase fava beans only when they are more prominent in markets. Addressing local challenges to legume production and consumption can pave the way for effective interventions to increase the intake of these sustainable foods. This study suggests promoting fava beans as a locally cultivable option, which could reduce reliance on imports and enhance regional agricultural output. Interviewees suggested using targeted promotional tactics, such as short videos, cooking demonstrations, and influencer marketing on social media, as effective means to boost fava bean consumption. **Conclusions:** These exploratory findings indicate that such strategies may foster a more positive perception and integrate fava beans into everyday diets in the region.

## 1. Introduction

Legumes are nutritionally rich, offering high-quality protein, complex carbohydrates, dietary fiber, and essential micronutrients such as iron, magnesium, potassium, and B vitamins [1]. Among over 16,000 legume species, fava beans (*Vicia faba* L.) rank third in global production, following soybeans and peas [2]. Beyond their nutritional value, legumes contribute to environmental sustainability through nitrogen fixation, improving soil fertility, and reducing dependence on chemical fertilizers [3]. Fava beans are particularly promising for European agriculture, with yields reaching up to 8–9 tons ha^−1^ in some regions [4].

While sharing the core nutritional benefits of legumes, fava beans present distinct advantages. They are not classified among the major food allergens by the United States Food and Drug Administration. In addition, recent evidence suggests a comparatively lower allergenic potential than soy and peanuts [5]. They also offer a higher protein content, ranging from 19.8% to 30.9%, compared to chickpeas (17–22%), peas (20–25%), and black beans (22–26%) [6,7,8]. As a potential downside, fava beans contain pyrimidine glycosides that protect the plant against pests; these are vicine and convicine, compounds associated with favism that may hinder consumer acceptance. Fortunately, the severe form of this condition is rare [9], and various food processing methods and breeding approaches have been shown to significantly reduce or eliminate these compounds [10,11].

At an industrial level, fava beans can be transformed into a variety of functional ingredients, including protein isolates, flours, and concentrates [12]. Additionally, fava bean extrudates have demonstrated effectiveness when added to meat-based products [13]. Fava beans possess beneficial natural properties such as gelation, emulsification, and the ability to absorb water and fats. Furthermore, they can be part of the gluten-free and plant-based food market, making them a valuable resource for the food processing industry, given their wide range of applications [14].

Legumes are a key component of the agro-ecosystem in the Mediterranean region [15], where diets tend to prioritize food consumption with seasonal production [16]. The Mediterranean dietary pattern prioritizes plant-based foods, including cereals, legumes, nuts, fruits, and vegetables, while reducing the intake of red meat [17]. The Iberian Peninsula follows these Mediterranean diet patterns [18]. Within the context of the Mediterranean diet, it is recommended to consume legumes at least three times a week, and in Portugal, the recommendation is to consume daily 1 to 2 portions of 25 g in dry weight or 80 g fresh/cooked legumes [19]. This practice is linked to a reduced risk of various metabolic health conditions, including obesity, cardiovascular disease, and type 2 diabetes [20].

However, fava beans are not among the most consumed legumes in the region; Iberian and vegetarian/vegan consumers alike seem to indicate a greater preference for beans, chickpeas, and lentils [21]. In Spain, several legumes grown in the territory display quality and origin labels such as “Protected geographical indication” (PGI), which is the case for Faba Asturiana, Faba del Lourenzá, and Garbanzo de Escacena, among others. All this to showcase the dietary link and soil quality of their longstanding traditions [22].

There has been a per capita decline in legume consumption, with an estimated intake of 9.2 g of legumes per day in Spain [23]. In Portugal, estimates indicate approximately twice this level, around 18 g per day [24]. Spain’s levels (9.2 g/day) fall below the WHO recommendations of at least 15 g/day, and studies indicate that this consumption mainly involves beans and peas [25,26]. There is limited data on fava bean consumption in the Iberian Peninsula, but it is assumed that they are consumed, given the presence of some traditional dishes featuring fava beans. Even among vegetarians and vegans, who often consume higher amounts of legumes and nuts [27], it remains unclear whether fava beans are frequently included in their diets or if their legume choices are more diverse than those of non-vegetarians, even though vegetarian consumers might be willing to try less common foods like fava beans to replace products they have eliminated from their diets [28].

Case studies have successfully developed fava bean-based food products, such as bread, pasta, and meat analogs [29,30], but somehow, they do not seem to be replicated at an industrial level.

Several European projects have worked towards an increase in the consumption of legumes [15,31]. They seek to valorize legumes for their farm benefits, breed more adapted varieties, and develop novel food products with legumes, aiming to highlight the potential of including local varieties and landraces in local contexts. However, for fava beans to reach local markets and promote consumption, all supply chain elements, including local production, harvesting, processing, storage, distribution, and retail presence, must be connected. Policies that promote the development of legume-based systems are often in disagreement with other factors that influence stakeholders’ decisions, such as social, structural, and behavioral factors [32].

The objective of this study is to identify the existing obstacles to the adoption of fava beans in several given social settings. It aims to reveal the most essential actions to take to simplify the resolution of these barriers.

## 2. Materials and Methods

### 2.1. Study Design

Qualitative research was selected as the preferred method to implement for this small-scale study because it enables a comprehensive exploration of the perceptions and attitudes that influence the consumption of specific foods. In line with other qualitative nutrition research, the study was designed to explore expert perspectives on barriers and opportunities for fava bean consumption, rather than to estimate population-level behaviors [33,34]. Sample size was determined by thematic saturation rather than statistical power, with a purposive sample recruited until no substantially new themes emerged in additional interviews [35].

The information collected helped to identify patterns and insights related to human behavior [36]. For this, various stakeholders were identified and invited to participate in one-on-one interviews to gain a better understanding of the factors that encourage or hinder fava bean consumption in the Iberian Peninsula. The aim was to create a relaxed atmosphere where each stakeholder could openly express their beliefs -through semi-structured interviews guided by pre-established topics- without time constraints, drawing from their professional experiences, backgrounds, family dynamics, and geographical locations to enrich the discussion.

### 2.2. Participants and Recruitment

The participants (*n* = 14) were identified by purposive sampling based on their professional activities, country of origin, and current residence. Exclusion criteria included subjects who did not reside in Portugal or Spain, who could not be interviewed in English, Spanish, or Portuguese, or whose professional background did not belong in the agri-food value chain (farmers, retailers, nutritionists, catering professionals, and consumers). Within each profile, participants were approached because their current role provided direct experience with legume production, marketing, or consumption. Additionally, this selection criterion ensured diverse and relevant perspectives for capturing the core challenges and motivations behind fava bean consumption in the Iberian Peninsula.

Some qualitative studies suggest that 10–15 interviews could be enough data to formulate key themes of a topic [37]. Consistent with the focus on depth rather than statistical representativeness, this study comprised a final sample of 14 participants across key profiles (*n* = 7 from Portugal and *n* = 7 from Spain). A high degree of redundancy in the responses was obtained when conducting the final interviews, a circumstance that suggests that saturation had been reached. There were no rejections to the interview requests performed for this study [35].

A comprehensive, cross-profile analysis, including interviews with nutritionists, retailers, farmers, catering professionals, and both vegetarian and non-vegetarian consumers in Portugal and Spain, was undertaken to compare how professionals with similar roles in each country perceived barriers and opportunities for fava bean consumption without aiming for demographic representativeness.

They were recruited via email invitations, where they were informed beforehand of the study’s purpose. All participants provided signed consent to be interviewed. This study was endorsed by the Institute of Bioethics at the Catholic University of Portugal through the Ethics Screening Report 11/2017. Table 1 summarizes the demographic data of the participants.

### 2.3. Data Collection

The interviews took place between December 2023 and January 2024. All interviews were conducted by the same researcher, in one-on-one sessions that were predominantly video recorded over a video conference platform (*n* = 12). When the interviews took place in person (*n* = 2), they were recorded with the aid of a mobile device. The sessions had a duration of 20–120 min, reflecting the time each participant needed to address the core topics and provide examples from their experience. No limits were set in terms of the interview duration or deviation from the topic to make the participants feel at ease, to create a relaxed environment, but most importantly, to grant freedom in terms of their perceptions and thoughts about each topic. Despite this flexibility, all interviews followed the same semi-structured guide and covered the main question areas before being concluded.

#### Interview Questions

Demographic information for each participant was collected. The semi-structured guide for interviews included six categories of topics, the aim being to delve into their own experiences towards each of the categorized topics (general knowledge of legumes, perceptions of legume production, strategies for increasing consumption rates, feelings and emotions related to fava beans, compliance of dietary guidelines (or dietary compliance, including consumption frequency) and processing and exploitation). While one question used a dichotomous format (‘Do you know any typical dishes…’) as an entry point to discussion, all responses were systematically followed by open-ended inquiries requesting specific examples, festive contexts, and comparisons with other celebrations in order to generate qualitative data on cultural associations [38]. Table 2 presents the interview guide, which was verbally presented to each subject to gain insight into the reasons behind their opinions and behaviors concerning fava beans. The interviews were conducted in the interviewee’s native language, Portuguese or Spanish, to provide a more relaxed space for the interviewee to be able to express thoughts that could otherwise be hindered in subjects with poor English command.

### 2.4. Data Analysis

**Figure 1:**
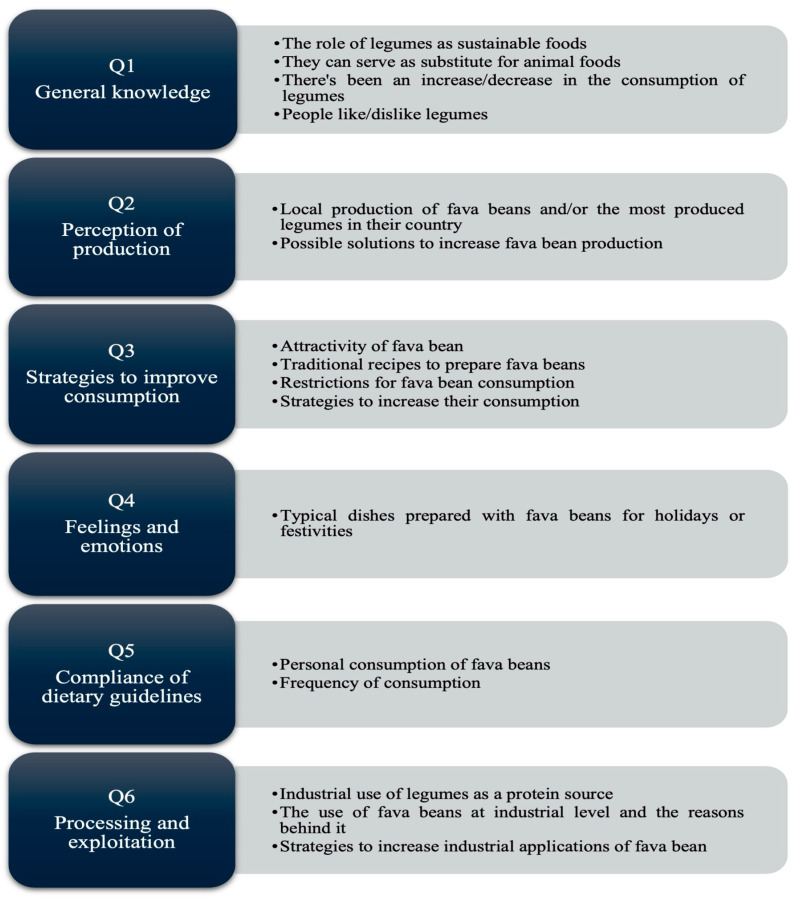
Categories and subcategories from the interview questions and statements.

The answers were transcribed into text with the transcription software Cockatoo (v3), Cockatoo, Philadelphia, PA, USA. The artificial intelligence (AI)-generated transcriptions were then corroborated by the researchers to ensure that no data had been lost during the process of transcription. All interviews were translated into English, once again by an AI tool, Google Translate (version not specified), Google LLC, Mountain View, CA, USA. Data were then analyzed using a qualitative content-thematic approach, involving open coding of transcripts, grouping similar codes into subthemes and organization of related subthemes into broader themes [39]. At first, a free-floating reading led to the elaboration of an information matrix on a spreadsheet (Microsoft Excel V 16.93), which allowed for seeing and comparing all the interview’s information to create a structure for the pre-analysis of data. Using this matrix, the statements given by each subject were categorized into the six interview topic categories (Table 2) initially established, to later create subcategories from each topic, based on the obtained information that could respond to this study’s research objectives (Figure 1). This process involved inductive coding, whereby subcategories and themes were generated from the data itself rather than pre-established. Similar codes were then grouped into subthemes, and related subthemes were organized into broader themes, with constant checking back to the transcripts to ensure that each theme accurately reflected all underlying data. Coding was updated repeatedly after each set of interviews to monitor thematic saturation, and by approximately the twelfth interview, no new codes were being identified within the main topic areas, with the last two interviews confirming existing themes. Subthemes were retained when they were clearly distinct, directly linked to the research questions and supported by multiple participants across profiles, following recommendations for thematic analysis in qualitative health research [40,41,42]. Secondly, the interviews were analyzed in depth to codify the text that belonged to each of these already established sub-categories. Lastly, the results were interpreted by merging data from the theoretical concepts with the obtained empirical data.

## 3. Results and Discussion

### 3.1. Demographics of Interviewees

The participants of this study ranged between the ages of 25 and 60. They were predominantly women (87%), which may reflect greater involvement in culinary domains, and their professional occupation was the most important criterion for selection, followed by their country of origin (Table 1).

### 3.2. Opinions About Fava Bean Production and Consumption

The interview questions (Table 2) guided discussions toward recurring subcategories that informed the results. The general knowledge category (Q1) explored whether legumes are perceived as sustainable protein alternatives to meat and whether this perception influences their consumption. Perception of production (Q2) focused on interviewees’ awareness of legume production, specifically fava beans, and their suggestions for improving local cultivation. In strategies to improve consumption (Q3), participants discussed their interest in fava beans, recipe knowledge, and reasons for avoiding them, offering initial ideas to boost consumption. The category of feelings and emotions (Q4) examined whether emotional connections or memories could encourage consumption on specific occasions. Dietary compliance (Q5) assessed the regularity and consistency of fava bean intake. Lastly, processing and exploitation (Q6) explored market opportunities and industry perspectives on integrating fava bean products into retail. Collectively, these subcategories revealed what participants perceived to be the key barriers to fava bean consumption in the Iberian Peninsula and allowed interviewees to propose actionable solutions for increasing their dietary presence.

These discussions provided valuable information on the reasons behind the low consumption rates of fava beans in the region. The structured questions served as a starting point for a conversation that aimed to dig deeper into the development of specific topics.

#### 3.2.1. General Knowledge

This section presents the gathered responses to Q1 regarding participants’ general knowledge and their perceptions of legumes broadly. Interviewees expressed two main perspectives on legumes, recognizing them as healthy and sustainable foods. In Portugal, participants linked legumes to environmental benefits through their role in plant-based diets (P1: “There is more people every day who follow a plant-based diet”) and noted that not everyone enjoys eating them. They suggested that more versatile preparation methods could increase interest. Despite this, they felt legume consumption was rising. In Spain, consumption was more rooted in tradition, with a strong desire to preserve ancestral dishes (S3: “Our grandmothers prepared lentils and chickpeas and that has been passed on as something nutritious that we should implement in our diets”). This cultural heritage is seen as a key reason why many Spaniard participants still enjoy legumes. Catering professionals in both countries (P7 and S7) noted the challenges of preparing legumes in large quantities, citing long preparation times and common complaints about digestive discomfort or the absence of animal protein. While the Spanish participants believe legume consumption is declining, both Portuguese and Spanish respondents agreed that legumes are rarely seen as true substitutes for animal protein. Instead, they are often served with meat. Although scientific evidence supports replacing animal protein with legumes, especially when paired with cereals [43,44], this idea is still met with skepticism. Expert’s opinions from Spain, aligned with actual data: legume consumption dropped by 2.3% compared to 2022 [23].

Legume consumption is more established in the 65+ age group, as data shows that the consumption of legumes for this age cluster in a year is 5.4 kg per capita, a higher value when compared to the average yearly consumption per capita of 3.3 kg [23]. Another study shows that legumes are indeed consumed along with processed meats, potatoes, and other ingredients that might be high in sodium, and yet, there was no association with unhealthy aging [45]. Lastly, a study with Portuguese consumers shows that they are indeed willing to transition to plant-based diets or to at least include more legumes in their meals; However, these decisions depend largely on the sociodemographic background of each person. It is expected to see more willingness to include legumes as protein alternatives in women than men, as well as in people with higher levels of education [26].

#### 3.2.2. Perception of Production

To assess stakeholder knowledge of the legume supply chain, specifically fava beans, participants were asked which legumes they believe are most produced in their country and whether fava beans were among them. Most respondents from both Portugal and Spain were unsure, speculating beans for Portugal and chickpeas for Spain. Only farmers (P2 and S5) confidently named specific legumes. Regarding fava beans, most stakeholders were unaware of their local production but assumed they might be grown in Portugal (P7: “I know they’re produced because I know many people who plant them in their gardens”) and in Spain (S2: “[for fava beans] there is so little demand that we have enough with the national production”).

When asked how production could be increased, interviewees from both countries agreed that low demand limits incentives. Therefore, monetary incentives, academic research, or more significant campaigns to promote fava bean consumption could increase the demand for this legume. While the perception of low production was widespread, actual data from 2022 shows otherwise: Portugal produced 3700 tons and Spain 43,610 tons of fava beans [2]. This perception may be skewed when compared to green pea production, which reached 8030 tons in Portugal and 109,980 tons in Spain [2]. Fava beans are cultivated across Asia, Africa, and the Mediterranean, including Portugal and Spain, thanks to their adaptability to various climates, even boreal ones [46]. Several EU projects aim to promote their cultivation, exploring uses beyond human consumption, including applications as protein isolates, fiber sources, gluten-free bases, animal feed, crop rotation tools, and ingredients in plant-based and novel food products [21,47].

#### 3.2.3. Strategies to Improve Consumption

Fava beans are generally perceived as one of the less attractive legumes. Some Portuguese participants noted that they are mostly consumed by older individuals and often prepared with animal protein (P4: “[fava beans] is the one that people like the least. I believe peas and fava beans are the most difficult to introduce, and what [people] normally like is beans, chickpeas, and lentils”). Another perspective highlighted a lack of familiarity despite potential (P6: “We [vegetarians] cook a lot of recipes using legumes, but since the nutritional profile of fava beans is the same as the other legumes, we tend to go for those [legumes] that we are more familiar with”). This reflects a broader issue: the unique qualities of fava beans are not widely known. Promoting their sustainability was suggested as a strategy to boost interest. As previously discussed, fava beans offer several advantages: they contain more plant-based protein than chickpeas or beans [6], are free from common allergens like soy and peanuts, naturally fix nitrogen into the soil [3], require fewer pesticides, grow well in the Mediterranean and much of Europe [4,15,22], and have promising industrial applications [12,13].

Experts from Spain associated fava beans strongly with winter (S4: “I think we tend to associate them [fava beans] with high-calorie and heavier dishes”) and are often featured in seasonal stews and soups (S7: “We like them very much [fava beans] in a broth and mixed with vegetables. Towards areas of La Mancha and Castilla they usually make stews [with them]”). In Portugal, nearly all interviewees reported consuming fava beans in the same traditional dishes (with sausage or poached eggs), while in Spain, recipes varied regionally, ranging from tapas and paella to stews and scrambled eggs. This culinary diversity may enhance their appeal in Spain, where local traditions help keep fava beans on the table and part of cultural heritage.

When asked about any restriction for the consumption of fava beans, nobody could name a reason to avoid them, such as an allergy or intolerance; however, all the nutritionists interviewed in both countries, reported that fava bean consumption could provoke some flatulence or intestinal disturbance, which is an important consideration to make for certain groups of people, specifically those who follow a low fermentable oligo-, di- and monosaccharides, and polyols (FODMAP) diet [48]. A single interviewee spoke about favism (S2). However, he immediately explained that this was a rare condition and that favism should not be a reason to avoid fava bean consumption [9].

To boost fava bean consumption, stakeholders in both countries emphasized the importance of leveraging social media and internet trends. In Portugal, one participant even suggested introducing a new school subject focused on healthy eating and agriculture. School garden initiatives, incorporating nutrition education, family involvement, and hands-on gardening, have been shown to improve children’s fruit and vegetable intake and BMI [49]. However, some studies highlight challenges, such as school staff’s limited knowledge and the lack of sustained support without expert guidance [50], underscoring the need for public health policies that strengthen educational capacity. Spanish interviewees proposed collaborating with popular social media chefs, promoting air fryer-friendly recipes, and using novel kitchen tools (S3: “Influencers could educate people on social media or these chefs, who are like leaders of these dietary change movements, and such. They could show dishes prepared with fava beans, and from there, (…) educate people and create new recipes, because often it is simply that you do not know how to cook them”). Another suggestion was to enhance fava bean palatability through breeding (S2: “Developing varieties with lower tannin content or with a better flavor (…) like the same thing that was done for common bean”). Although information is limited, recent research links lipid oxidation to the bitter taste and beany aroma of fava beans [51]. While dehulling removes up to 92% of phenolic compounds, including tannins [46], breeders are now exploring genetic pathways to reduce lipid oxidation and saponin biosynthesis, which could lead to more appealing varieties [51].

It was an interesting contrast to see how younger interviewees (P1 and S1) were more inclined to propose internet-based campaigns to attract consumers towards fava bean consumption, while older interviewees (P3 and S2) resorted to proposing social engagement activities, such as creating a fava bean association or developing show cooking activities. Participants reported that traditional campaigns seem unable to deliver the message or attract enough attention from younger consumers. Strategies must be directed to audiences who are willing to change their current habits or to at least experiment with new foods. Studies show how social media (the most suggested strategy to implement among interviewees) can achieve a change in the user’s behavior and motivate them to perform small and concrete actions [52]. This opens the door for the implementation of projects or campaigns that aim to increase interest in fava beans, whether it is for consumers, retailers, or farmers. A study conducted on Meta’s social media platforms showed that a post displayed 7.5 million times, reached 2.5 million individuals, and generated 250,000 active engagements, proving that paid advertisement on these platforms is effective in terms of reach and cost [53]. Table 3 synthesizes cross-cutting strategies identified across participants, summarizing themes described in this section, rather than listing individual responses.

#### 3.2.4. Feelings and Emotions

In Portugal, participants reported no fava bean dishes tied to specific holidays or seasons, though some associated them with winter comfort food (P3: “I don’t associate fava beans with any holiday, but it does remind me a lot of a comfort dish”). In contrast, Spanish participants quickly identified traditional fava bean dishes linked to winter (see Section 3.2.3), such as winter paellas made with artichokes and fava beans, and regional stews like the Galician broth (S7: “The Galician broth always has favas (…) and for the day of Rosalia de Castro, we make ‘Caldo de Gloria’ that also comes with fava beans”). This seasonal and emotional connection suggests a strategy for promoting fava bean consumption. Research shows that holiday foods carry emotional weight, often associated with nostalgia and celebration. Given that much of the weight gained over the winter holidays tends to persist, due to increased social events, stress, and high-calorie meals [54], fava beans could serve as a healthier alternative during these times.

Another factor influencing fava bean consumption is seasonal availability: in the Iberian Peninsula, fava beans are harvested from March to September, with green beans ready 110–130 days after sowing [55]. This may explain their limited visibility in winter and reinforces their association with specific holidays rather than an everyday meal. Targeted interventions include frozen preservation for year-round access to fava beans, canned, dried, or flour processing of fava beans to extend usability, as well as policy support for farmers during off-season time periods that encourage fava bean cropping.

This subcategory explored the emotional comfort tied to certain foods. Comfort food was defined in 1997 as “food prepared traditionally with a nostalgic or sentimental appeal” [56]. It is especially satisfying for high-emotional eaters [57]. Promoting fava beans as a traditional, comforting dish is suggested as an intervention that could help integrate them more deeply at a greater scale into Iberian food culture, shifting their role from seasonal to a year-round staple. Table 4 summarizes recurrent themes that emerged across interviews and does not represent quantitative frequencies.

#### 3.2.5. Dietary Compliance

Participants from both countries reported consuming fava beans less frequently than they would like (S6: “[eating fava beans] Many times during the season, because as we harvest them and they stay fresh, we eat maybe twice a week. But of course, it’s only two months a year”). Some stakeholders harvested their fava beans, allowing for regular seasonal consumption, while others extended availability by freezing their supply (P2: “[when in season] we tend to eat many times, basically until we finish our supply, but after, I have to confess that I don’t buy any more”). These health awareness claims could have been potentially amplified by having 87% female participation in these interviews. A notable contrast emerged between the Portuguese (P4) and Spanish (S4) nutritionists interviewed: the former consumed fava beans only once a year, while the latter included them at least twice a month.

Despite the prevalence of healthy eating messages on social media and in public spaces, their effectiveness remains questionable (see Section 3.2.3). A recent study revealed that while regional campaigns in Andalucía, Madrid, and Galicia promoted consistent dietary messages, they often lacked clarity, especially regarding portion sizes and recommended frequency, limiting their practical impact. Consequently, 68% of the evaluated visual materials were deemed ineffective, as they did not convey the intended message [58]. This is just an example of many campaigns that have stopped being effective, and the need to create different approaches. Interviewees recognized that they often forget that fava beans are in their freezers, which leads to believe that they could be more present at the table if there were more exposure and out-of-the-ordinary campaigns in our surroundings.

#### 3.2.6. Processing and Exploitation

Regarding the industrial use of legumes, the majority of participants from both countries were aware that legumes are used in processed food products. In Portugal, participants mentioned they were not familiar with any specific industrial products made with fava beans, though they would not be surprised if such products existed (P3: “Fava beans could be used, but, indeed, what you see in the formulations of such products is mostly pea, soy, and even chickpea”). Spanish interviewees reported checking ingredient labels regularly and were able to provide concrete examples of existing products containing lentils, rice, chickpeas, and peas.

The Portuguese vegetarian participant (P6) was more familiar with pea- and rice-based meat analogs, while the Spanish vegetarian participant (S6) had more experience with soy- and pea-based alternatives. No participants mentioned having seen a food product made with fava beans. When asked why this might be the case, Spanish experts pointed to the low production levels of fava beans as a limiting factor, both in terms of supply chain stability and raw material cost. Concerns were also raised about the technological properties of fava beans and how they might behave during processing (S3: “I think that the supply chain is not yet big enough to supply the companies with tons or kilos. Supply could be the reason (…), but then, if they want to go and make a product with it and be able to meet the demand, they do not reach it because there is not yet the production line they need. It has no established quality parameters, so it is always the same flour. Once a company decides to use that ingredient, it needs consistency in its properties and that it is always homogeneous and such”).

Participants from both countries suggest that increasing awareness of fava beans’ versatility and benefits could help boost their consumption. They suggested that developing more recipes and showcasing them, particularly through social media and internet influencers, could help popularize fava beans (see Section 3.2.3 and Section 3.2.4). Increased consumer interest could, in turn, drive up production volumes, lower raw material costs, and encourage broader industrial use. While numerous studies have focused on the development of fava bean-based products [11,12,59], the advances in fava bean processing have yet to reach the food industry at the same scale as other legumes. One of the main challenges identified is the off-flavor and color imparted by fava bean ingredients, which are seen as undesirable in food formulations. However, these sensory characteristics can be improved through techniques such as germination, fermentation, dehulling, soaking, and/or heat processing [60]. Although effective, these pre-treatment steps add extra time and cost, two critical factors in industrial food production.

### 3.3. Strengths and Limitations

A key strength of this study lies in its cross-national comparison of expert and stakeholder perspectives from Portugal and Spain, enabling the identification of both commonalities and contrasts within the Mediterranean context. Conducting interviews in participants’ native languages facilitated more natural, uninhibited responses. Additionally, the inclusion of stakeholders from across the food system, from production to consumption, offered a holistic understanding of how these participants perceive societal attitudes towards fava beans.

However, limitations include the relatively small number of participants and the underrepresentation of certain professional profiles within the food value chain, which may have restricted the diversity of viewpoints. The sample was predominantly female (12 F:2 M), which may reflect greater participation rates among women in agri-food professional networks but could have influenced perspectives on culinary and consumer preferences. The purposive profile-based strategy and the use of online interviews may also have favored participants with higher digital literacy and, potentially, higher educational attainment, which could be a source of selection bias. The exclusion of non-consumers, due to self-selection bias, may have limited the scope of perspectives. Furthermore, the concise interview guide and the online format, which required a certain level of digital literacy, may have constrained participation and thematic depth. Despite these constraints, the study yielded rich and meaningful insights into the views of the interviewed stakeholders, which can help design future, larger-scale quantitative studies.

## 4. Conclusions

This exploratory study suggests that fava beans remain marginal in everyday eating practices in Portugal and Spain, according to interviewed stakeholders, revealing that despite a general appreciation for the legume, it remains largely overlooked in the reported daily diets of the region. Participants across the food value chain expressed appreciation for fava beans but acknowledged their infrequent consumption, often citing limited culinary familiarity and visibility.

From the perspective of interviewees, findings indicate that context-specific strategies may be needed. In Portugal, culinary innovation, digital engagement, and school-based food education appear promising for increasing consumption, whereas in Spain, where fava beans are already tied to winter and regional traditions, reinforcing their role as comfort food and linking them to seasonal celebrations may encourage a steadier consumption throughout the year.

The study highlights perceived psychological and cultural barriers, such as low visibility, limited culinary diversity, and entrenched food habits, providing a qualitative foundation for future research on legume adoption. From a practical standpoint, these insights can inform stakeholders interested in promoting legume consumption. Strategies such as innovative marketing, social media engagement, product reformulation, and collaboration with influencers and chefs could foster greater consumer interest. Enhancing supply through the development of palatable, consistent varieties may also support industrial uptake. Overall, the results should be interpreted as hypothesis-generating and as a starting point for larger, survey-based studies that can test these patterns in broader populations and additional countries.

## Figures and Tables

**Table 1:** Interviewees Demographic Characteristics.

Identifier	Country	Gender	Age	Stakeholder Type
P1 *		F	26	Consumer (<35 years)
P2 *		F	39	Consumer (>35 years)
P3 *		F	43	R&D at a food company
P4 *	Portugal	F	26	Nutritionist
P5 *		F	50	Farmer
P6 *		F	32	Vegetarian
P7 *		F	31	Catering nutritionist
S1 **		F	28	Consumer (<35 years)
S2 **		F	60	Consumer (>35 years)
S3 **		F	42	R&D at a food company
S4 **	Spain	M	31	Nutritionist
S5 **		M	54	Farmer
S6 **		F	31	Vegetarian
S7 **		F	28	Catering nutritionist

* Portuguese participant; ** Spanish participant; F—female; M—male; R&D—research and development.

**Table 2:** Interview topics.

Category	Statement
Q1 General knowledge	In recent years, much has been said about the role of legumes as healthy and sustainable foods. It has even been proposed that legumes can serve as a substitute for animal foods. In your opinion, have people adhered to these messages, increasing the consumption of legumes? What is your perception of the consumption of legumes in the Portuguese/Spanish population?
Q2 Perception of production	Regarding fava bean production, do you know if it’s widely produced in our country? Which are the most produced legumes in Portugal/Spain? In your opinion, what could be done to increase fava bean production in Portugal/Spain?
Q3 Strategies to improve consumption	Among legumes, what do you think is the popularity level of fava beans? Is it a legume appreciated by the population? Are there many Portuguese/Spanish recipes prepared with fava beans? In your opinion, what could be done to increase the consumption of fava beans in your country?
Q4 Feelings and emotions	Do you know any typical dishes or specialties made with fava beans that are consumed at festive times (e.g., Christmas)?
Q5 Dietary compliance (consumption frequency, processing, and exploitation)	Do you usually consume fava beans? If so, how? Can you tell how often you use them?
Q6 Processing and exploitation	Some industrialized foods use legume protein as a source of plant protein. Do you have any idea if fava beans are usually used in this type of production? If not, why not? What could be done to increase the use of fava beans as a protein source?

**Table 3:** Strategies to improve fava bean consumption.

Category	Proposal
Marketing	Social media campaigns, collaborations with food influencers and chefs, paid advertisements on social media platforms, online recipe sharing.
Culinary approach	Develop recipes using novel cooking tools, introducing modern cooking methods, new product development, on-site supermarket advertising and promotion.
Education	Implementing school programs on food literacy, integrating fava beans into school gardens and cooking workshops.
Scientific improvement	Genetic enhancement of fava beans (with low-tannin varieties) as well as producing more scientific evidence on the nutritional and environmental value of fava beans.
Industry support	Increasing supermarket presence, working with retailers to promote fava beans and encourage local farming.

**Table 4:** Strategies to improve fava bean consumption through feelings and emotions.

Category	Proposal
Link to holiday traditions	Leverage the already existing fava bean recipes in Spanish winter dishes (e.g., Caldo de Gloria, winter paella, Galician broth) and incorporate them into Portuguese traditions.
Comfort food	Create a focal point on the nostalgic and emotional appeal of fava beans during the cold, winter months.
Develop holiday recipes	Modify or improve the already existing festive meals to include fava beans.
Create all-year availability	Encourage industrial strategies to preserve fava beans beyond their natural harvest season.

## Data Availability

The data presented in this study are available on request from the corresponding author. The data are not publicly available due to ethical reasons.
